# Maternal separation and TNBS-induced gut inflammation synergistically alter the sexually differentiated stress response in rats

**DOI:** 10.1186/s13030-022-00258-x

**Published:** 2023-02-25

**Authors:** Ryoko Hasegawa, Kumi Saito-Nakaya, Li Gu, Motoyori Kanazawa, Shin Fukudo

**Affiliations:** 1 Department of Behavioral Medicine, Tohoku University Graduate School of Medicine, 2-1 Seiryo, Aoba, Sendai 980-8575 Japan; 2 Tohoku Medical Megabank Organization, Tohoku University, 2-1 Seiryo, Aoba, Sendai 980-8575 Japan; 3 Department of Psychology, School of Humanities and Management, Guangdong Medical University, Dongguan, China

**Keywords:** Attention-deficit/hyperactivity disorder, Corticotropin-releasing hormone, Early-life stress, Irritable bowel syndrome, Maternal separation, Visceral hypersensitivity

## Abstract

**Background:**

Neonatal maternal separation (MS) has been used to model long-lasting changes in behavior caused by neuroplastic changes associated with exposure to early-life stress. Earlier studies showed that transient gut inflammation can influence the development of irritable bowel syndrome (IBS). A prevailing paradigm of the etiology of IBS is that transient noxious events lead to long-lasting sensitization of the neural pain circuit, despite complete resolution of the initiating event. This study characterizes the changes in behaviors and neuroendocrine parameters after MS and early-phase trinitrobenzene sulfonic acid (TNBS)-induced colitis. We tested the hypothesis that MS and gut inflammation synergistically induce (1) hyperactivity in male rats and anxiety-like behaviors in female rats and (2) activation of the HPA axis in female rats and deactivation of the HPA axis in male rats after colorectal distention (CRD).

**Methods:**

Male and female rat pups were separated from their dams for 180 min daily from postnatal day (PND) 2 to PND 14 (MS). Early-phase colitis was induced by colorectal administration with TNBS on PND 8. The elevated plus-maze test was performed at 7 weeks. Tonic CRD was performed at 60 mmHg for 15 min at 8 weeks. Plasma ACTH and serum corticosterone were measured at baseline or after the CRD. Analysis of variance was performed for comparison among controls, TNBS, MS, and MS + TNBS.

**Results:**

In male rats, the time spent in open arms significantly differed among the groups (*p* < 0.005). The time spent in open arms in male MS + TNBS rats was significantly higher than that of controls (*p* < 0.009) or TNBS rats (*p* < 0.031, post hoc test). Female rats showed no difference in the time spent in open arms among the groups. MS and gut inflammation induced an increase in plasma ACTH in female rats but not in male rats at baseline.

**Conclusions:**

These findings suggest that MS and gut inflammation synergistically induce hyperactive behavior or exaggerated hypothalamic–pituitary–adrenal axis function depending on sex.

## Background

Early life experiences are one of the factors affecting psychological and physiological development and may lead to significant alterations in emotion and stress responses in later life [1–5]. Maternally separated animals have been intensively studied for decades and have also served as models of psychopathology [6, 7]. Especially, maternal separation (MS) is a well-characterized model of early-life stress used to study anxiety and depression [3, 8]. Various results have also been reported regarding fearful/anxiety-related behavior after MS protocols, with most separation schedules increasing fearful/anxiety-like behavior [9–13]. However, different behavioral phenotypes have also been reported depending on MS protocols, with some studies reporting hyperactive behavior [14–17]. Moreover, MS in rodents resulted in less anxious and more hyperactive behavior, which was considered to resemble attention-deficit hyperactive disorder (ADHD) [18, 19]. The factors determining animal behavior after MS are largely unknown.

Irritable bowel syndrome (IBS) is a common disorder characterized by chronic abdominal pain associated with alterations in bowel habits in the absence of a major organic pathology [20]. The original view of the disease as a primary disturbance in the gut is being conceptually refined to include a complex and disordered interaction between the brain and the gut [21, 22]. IBS is triggered or exacerbated by psychosocial stress [22–24]. In addition, early-life stressors increase the risk of IBS [25]. Psychological and physiological stress experienced in early life activates and triggers functional changes in visceral functions and visceral sensitivity to noxious stimuli [26–28]. In particular, MS induces gut dysfunction [29–33]. Patients with IBS have a higher rate of anxiety, depression, and somatization [34]. Individuals with ADHD have more diagnosis of IBS with odds ratio 1.67 with 95%CI from 1.56 to 1.80 than those without ADHD [35]. Not only stress but also gut inflammation is likely a causative factor for IBS [36]. Prospective studies indicated that a substantial proportion of patients (3–36%) with acute bacterial gastroenteritis develop IBS symptoms [37, 38]. Earlier studies also clarified that recovered animals after the experimental colitis induced by trinitrobenzene sulfonic acid (TNBS) show exaggerated response to colorectal distention (CRD) [39, 40]. These studies suggest that there may be a behavioral link between stress-related disorders (e.g., IBS, ADHD, and anxiety/depression/somatization) and early experience of noxious stimuli (e.g., MS and gut inflammation).

A previous study by our group clearly demonstrated that IBS patients show an exaggerated response to administration of corticotropin-releasing hormone (CRH) [41]. Sensory and motor dysfunctions of the colon in IBS patients are improved by administration of a CRH antagonist [42]. Moreover, the electrophysiological properties of the brain in IBS patients are also dramatically normalized by administration of a CRH antagonist [43]. In an animal model of IBS, CRH receptor-1 (CRH-R1) antagonist reverses anxiety-like behaviors induced by CRD [44]. Combination of previous TNBS colitis and repetitive CRD makes the colon hypersensitive [45]. Another study demonstrated that CRH-R1 is primarily involved in the water avoidance stress-induced colonic motor response [46]. Neonatal rats exposed to MS demonstrate altered function in the hypothalamic–pituitary–adrenal (HPA) axis [47, 48]. An earlier study indicated that neonatal trauma induced phenotypic changes in adulthood, including increased permeability of gut mucosa to stress via mechanisms involving CRH [31]. Therefore, it is natural to assume that altered HPA axis tone is involved in MS and recovered inflammation.

The unsolved pathogenesis and pathophysiology of brain-gut disorders is related to the sex difference [49, 50]. IBS [49, 50] and anxiety/depression [51] are more predominant in female individuals. Most studies on depression have reported with female:male ratios of 2:1 [51]. Increasing evidence from limited studies supports similar prevalence rates for pain-related symptoms in IBS, but a greater female predominance in non-pain-associated symptoms of constipation, bloating, and extraintestinal manifestations [49, 50]. Evidence suggests that IBS symptoms are influenced by the menstrual cycle, with an amplification of symptoms during the late luteal and early menses phases [49, 50]. By contrast, developmental disorder is predominant in male individuals [52]. Although most studies investigating the long-term effects of MS have used male rodents, those that studied both males and females have found significant sex differences in the effects of MS [9, 53]. Male and female rats may differ in numerous neuroendocrine and behavioral parameters, and vulnerability to stress may be sex dependent [54–56]. Therefore, precise behavioral comparisons between male and female subjects are necessary for the accurate understanding of stress-related disorders as a typical manifestation of the biopsychosocial model.

In the present study, we focused on the behavioral and neuroendocrine changes induced by MS and neonatal colorectal inflammation. Based on previous findings, we tested our hypotheses that MS and gut inflammation synergistically induce (1) hyperactivity in male rats and anxiety-like behaviors in female rats and (2) activation of the HPA axis in female rats and deactivation of the HPA axis in male rats after CRD.

## Materials and methods

### Animals

Timed-pregnant female Wister rats (*n* = 21) on gestation day 16 were obtained from Charles River Breeding Laboratories, Japan. Upon arrival, the rats were singly housed in the same room, which was maintained on a 12-h light/dark cycle (lights on at 8:00 am). Parturition was checked once a day (9:00 am), and the day of birth was considered PND 0. On PND 1, litters were cross-fostered (to minimize effects of genetic or prenatal variability) and adjusted to 12 pups each with about the same numbers of male and female pups. All dams were given free access to food and water on a 12-h light/dark cycle (lights on at 8:00 am) at a temperature of 23 ± 1 °C. Experimental conditions were assigned per litter. The irritation procedure and the experimental testing were conducted during the light component of the cycle. This study was based on the Guide for the Care and Use of Laboratory Animals and approved by the Animal Ethics Committee of Tohoku University School of Medicine.

### Protocol

**Fig. 1:**
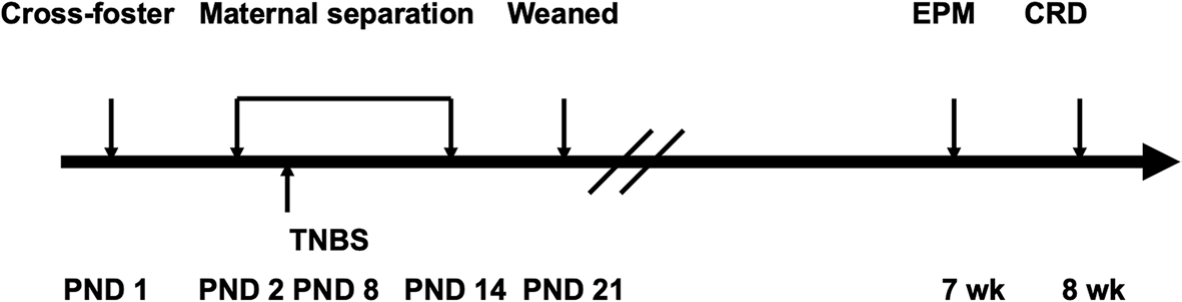
Experimental design. Maternal separation (MS) procedures were performed from postnatal days (PNDs) 2 to 14. On PND 8, rats were exposed to TNBS-induced colitis and allowed to recover. At 7 weeks of age, rats were exposed to the elevated plus-maze test. At 8 weeks, colorectal distention (CRD) was performed

The experimental design was shown in Fig. 1. Rats were randomly assigned to four groups: (1) no separation and no inflammation (Control), (2) no separation and inflammation (TNBS), (3) MS and no inflammation (MS), and (4) MS and inflammation (MS + TNBS). The pups with MS were separated from their dams during PND 2 to 14, whereas the pups without MS remained with their mothers. To induce inflammation, rats were treated with TNBS (WAKO Pure Chemicals, Ltd., Tokyo, Japan) on PND 8. For no inflammation, rats were treated with vehicle (50% ethanol). In the 7th week, rats were exposed to an elevated plus-maze (EPM) and their behaviors were monitored and quantified. In the 8th week, rats were exposed to CRD as a visceral stimulus. After CRD for 15 min, the rats were killed by decapitation. The basal blood of each group was obtained without CRD. Blood was collected in a tube and the plasma was separated by centrifugation at 3000 rpm and stored at –30 °C.

### Maternal separation

Litters of 12 pups were randomly assembled for the fostering. The MS protocol was essentially the same as in earlier reports [31, 57–59]. Briefly, rats were exposed to a 180-min period of daily separation from dams (MS180) on PND 2 to 14. Separation was loaded at 9:00 am ± 30 min each day. The dams were removed from the maternity cages and placed into separate identical cages until the end of the manipulation. At dam removal, MS180 litters were removed as a group from the nest, weighed, and placed as a group in an isolation cage in an adjacent room. The cage was lined with chip bedding and placed in a neonatal cage and kept at 37 ± 0.5 °C by using a heating mat (KN–474; Natume, Tokyo, Japan) set underneath the cage. At the end of the daily separation, the pups were returned to their maternity cage and rolled in the soiled home cage bedding material before being reunited with the dam. On PND 22, all rats were weaned and the litters were housed in individual cages by sex and in the same treatment pairs.

### Menstrual cycle

At the 7th week, the menstrual cycles of female rats were evaluated by a vaginal smear after Giemsa staining. On the basis of the microscopic reading of the smears, rats were subdivided according to the four phases of the cycle (proestrus, estrus, metestrus, and diestrus). Animal studies have shown increased responsiveness to greater autonomic manifestations during the metestrus/diestrus cycle phase, which corresponds to the perimenstrual phase in women [60, 61]. Therefore, all experiments in female rats were performed during the metestrus/diestrus phase.

### Previous inflammation

The inflammation model of experimental colitis used here is well documented elsewhere [39, 40, 45]. Briefly, TNBS was dissolved in 50% ethanol to a concentration of 120 mg/ml. At PND8 during maternal separation rats were transferred in the paper container for treatment. A polyethylene-60 catheter was inserted 3 mm past the anus to lie approximately at the level of the splenic flexure. Rats were infused with 1.5 mg/50 µl of the TNBS plus ethanol solution. Control rats were similarly incubated but infused with 50 µL of the 50% ethanol vehicle only. This procedure involves a 6-week recovery period.

### Elevated plus-maze test

Anxiety-like behavior was evaluated using a plexiglass EPM [44, 45]. The four arms (50 × 10 cm) were 1 m above the floor with a 10-cm center. The closed arms had 40-cm-high walls. Rats were placed in the center of the maze and were allowed to explore freely for 5 min; their behavior was recorded by video and using EthoVision software (TARGET system). The test belongs to the group of unconditioned anxiety models used for the development of putative anxiolytic compounds. The paradigm is based on rats’ innate aversion to open and high places.

### Colorectal distention stimuli

The experimental CRD procedure and visceral sensitivity testing have been extensively reported elsewhere [62] and we essentially used the same method. The rat was lightly restrained in a plastic tube. A polyethylene balloon of 2.5 cm in diameter was inserted into the colorectum via the anus. The distal end of the balloon was positioned 1 cm proximal to the anus and was secured in place by taping the balloon catheter to the base of the tail. The balloon pressure, which represents intracolonic pressure, was continuously monitored online with the aid of a computerized barostat system (G and J Electronics Inc., Toronto, Canada). The colorectum was distended at a pressure of 60 mmHg for 15 min. CRD in rats causes an easily monitored pseudaffective response: a contraction of the abdominal and hind limb musculature (i.e., a visceromotor reflex) [62, 63].

### Neuroendocrine function

Immediately after the rats were decapitated, blood was collected in chilled polyethylene tubes containing 200 µl (74.5 mg) of EDTA and separated with a centrifuge. The plasma was stored at –30 °C until analysis. Plasma adrenocorticotropic hormone (ACTH) and serum corticosterone were measured by radioimmunoassay. Some rats could provide less volume of blood for assay of ACTH and corticosterone. Thus, number of rats for HPA axis data was smaller than number of rats for behavioral experiments.

### Statistical analysis

All data are expressed as mean ± SE. A two-tailed Student’s *t*-test was used to analyze differences between two groups. When more than two groups were compared, the significance among groups was evaluated by three-, two-, and one-way analysis of variance (ANOVA), and further statistical post hoc comparisons were performed using a post hoc Tukey’s test. A probability level of < 0.05 was considered to be statistically significant. All statistical calculations were performed using SPSS for Windows (ver. 12.0 J).

## Results

### Elevated plus-maze test

**Fig. 2:**
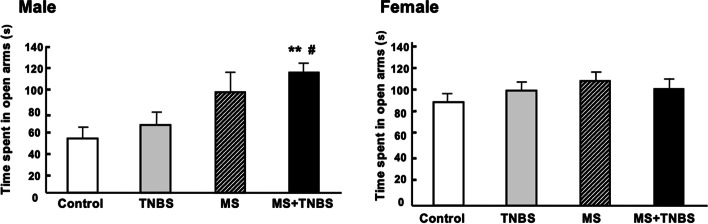
Time spent in open arms in the elevated plus-maze. Effects of early-life stress (MS) and/or previous inflammation (TNBS) on behavior are expressed as the mean ± SE. Control (male, *n* = 12; female, *n* = 12), TNBS (male, *n* = 16; female, *n* = 16), MS (male, *n* = 15; female, *n* = 11), and MS + TNBS (male, *n* = 15; female, *n* = 13). ^*^P < 0.05, ^**^P < 0.001 MS + TNBS vs. control. ^#^*P* < .05, ^##^*P* < 0.001 MS + TNBS vs. TNBS. left, males; right, females

**Fig. 3:**
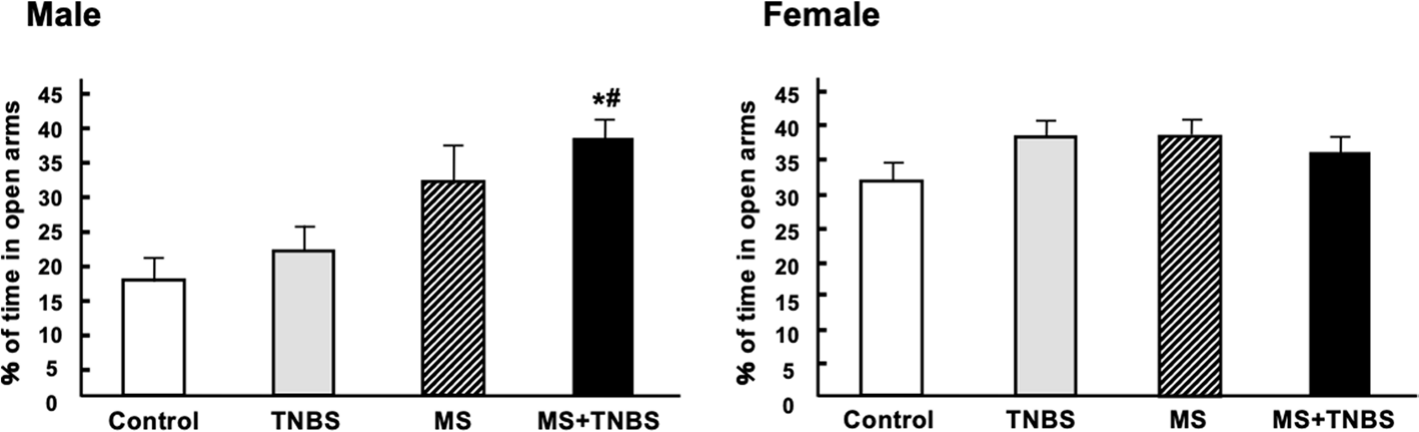
Percentage of time spent in open arms in the elevated plus-maze. See the legend of Fig. 3 for details. left, males; right, females

**Fig. 4:**
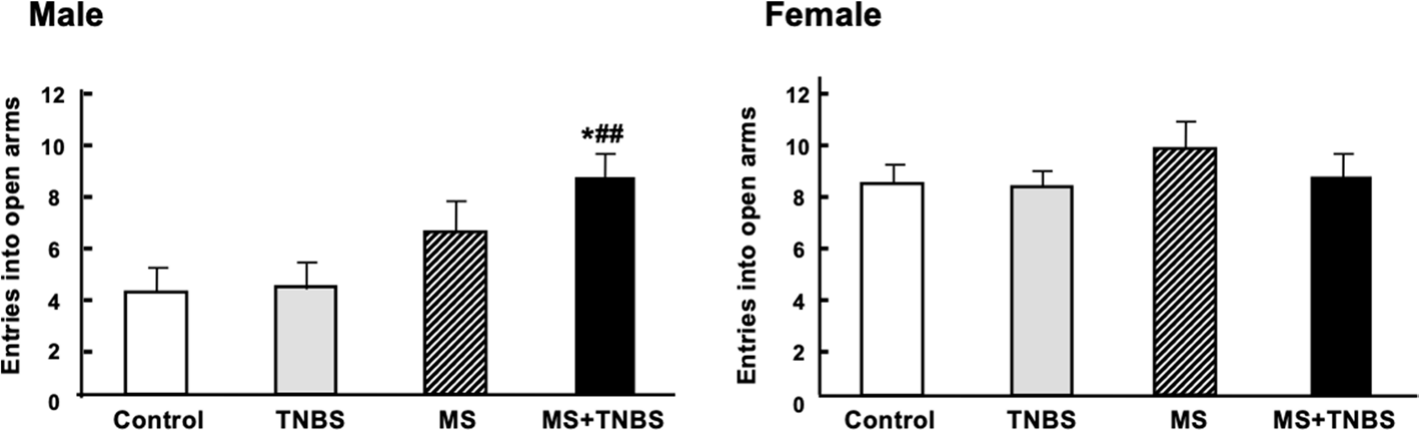
Entries into open arms in the elevated plus-maze. See the legend of Fig. 3 for details. left, males; right, females

**Fig. 5:**
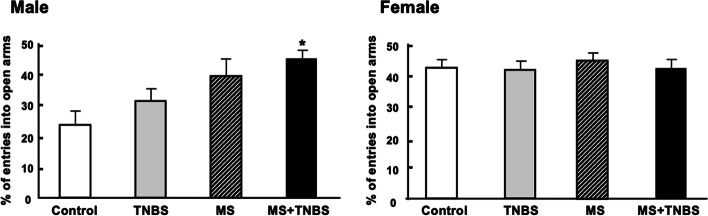
Percentage of entries into open arms in the elevated plus-maze. See the legend of Fig. 2 for details. left, males; right, females

**Fig. 6:**
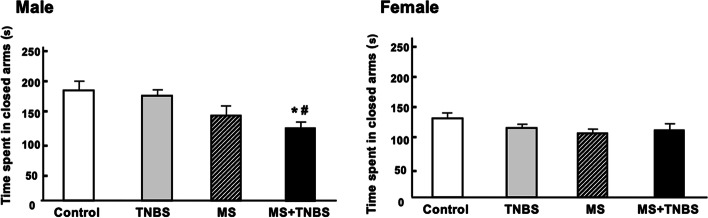
Time spent in closed arms in the elevated plus-maze. See the legend of Fig. 2 for details. left, males; right, females

Table 1 shows the results of a three-way ANOVA performed with each category as a dependent variable and with sex, MS, and TNBS as independent variables. The results indicate that, concerning the time spent in open arms, the factors sex, MS, and the interaction between sex and MS were significant. This pattern was also seen in the percentage of time spent in open arms and the time spent in closed arms, as confirmed by significant effects of the same factors and same interactions. Regarding the time spent in open arm entries, ANOVA revealed significant main effects of sex (F_1.102_ = 12.073, *p* < 0.001), main effects of MS (F_1.102_ = 11.228, *p* < 0.001), and a sex × MS interaction (F_1.102_ = 4.772, *p* < 0.031). Total travel distance of three-way of ANOVA revealed significant main effects of sex (F_1, 102_ = 8.243, *p* < 0.005) and MS (F_1, 102_ = 4.379, *p* < 0.038). The results of the anxiety-related behavior in the EPM performance are presented in Figs. 2, 3, 4, 5, 6. All analyzed variables of the behavioral response to the EPM, except entries into closed arms, differed significantly between male rats (control, *n* = 12; TNBS, *n* = 16; MS, *n* = 15; and MS + TNBS, *n* = 15) and female rats (control, *n* = 12; TNBS, *n* = 16; MS, *n* = 11; and MS + TNBS, *n* = 13). In male rats, the time spent in open arms significantly differed among the groups (ANOVA, F_3,54_ = 4.795, *p* < 0.005). The time spent in open arms by male MS + TNBS rats (112.1 ± 10.3 s) was significantly higher than that of controls (50.4 ± 10.3 s, *p* < 0.009) or TNBS rats (63.1 ± 11 s, *p* < 0.031, post hoc test) (Fig. 2). Female rats showed no difference in the time spent in open arms among the groups (Fig. 2). The percentage of time spent in open arms by male rats significantly differed among the groups (ANOVA, F_3,54_ = 4.793, *p* < 0.005). The percentage of time spent in open arms by male MS + TNBS rats (37.4 ± 3.4%) was also significantly higher than that of controls (16.8 ± 3.4%, *p* < 0.009) or TNBS rats (21 ± 3.7%, *p* < 0.032, post hoc test) (Fig. 3). Female rats showed no difference in the percentage of time spent in open arms among the groups (Fig. 3). Entries into open arms were shown in Fig. 4. The percentage of open arm entries of male rats significantly differed among the groups (ANOVA, F_3,54_ = 4.023, *p* < 0.012). Male MS + TNBS rats showed a significantly higher percentage of open arm entries (44.7 ± 3.3%) than controls (23.4 ± 4.7%, *p* < 0.011, post hoc test) (Fig. 5). Female rats showed no difference in the percentage of open arm entries among the groups (Fig. 5). ANOVA of the time spent in closed arms by male rats revealed a significant difference among the groups (F_3,54_ = 4.795, *p* < 0.007). Male MS + TNBS rats spent significantly less time in closed arms (119.5 ± 10.3 s) than controls (179.6 ± 15, *p* < 0.014) or male TNBS rats (170 ± 10.5 s, *p* < 0.03, post hoc test) (Fig. 6). Female rats showed no difference in the time spent in closed arms among the groups (Fig. 6).

**Table 1:** Three-way ANOVA results of the elevated plus-maze

Effects	df	F	*P*-level
(A) Time spent open arm			
Sex	**1**	**12.073**	0.001**
MS	**1**	**11.228**	0.001**
TNBS	**1**	**1.106**	**0.296**
Sex × MS	**1**	**4.772**	0.031*
Sex × TNBS	**1**	**0.673**	**0.414**
MS × TNBS	**1**	**0.052**	**0.821**
Sex × MS × TNBS	**1**	**0.34**	**0.561**
Residuals	**102**		
(B)% Time spent open arm			
Sex	**1**	**12.044**	0.001**
MS	**1**	**11.212**	0.001**
TNBS	**1**	**1.108**	**0.295**
Sex × MS	**1**	**4.782**	0.03*
Sex × TNBS	**1**	**0.669**	**0.415**
MS × TNBS	**1**	**0.052**	**0.82**
Sex × MS × TNBS	**1**	**0.337**	**0.563**
Residuals	**102**		
(C) % Open arm enties			
Sex	**1**	**10.204**	0.002*
MS	**1**	**7.751**	0.006*
TNBS	**1**	**0.815**	**0.369**
Sex × MS	**1**	**6.204**	0.014*
Sex × TNBS	**1**	**2.237**	**0.138**
MS × TNBS	**1**	**0.041**	**0.84**
Sex × MS × TNBS	**1**	**0.026**	**0.873**
Residuals	**102**		
(D) Time spent closed arm	**1**		
Sex	**1**	**19.356**	0.0001***
MS	**1**	**11.39**	0.001**
TNBS	**1**	**1.759**	**0.188**
Sex × MS	**1**	**4.178**	0.044*
Sex × TNBS	**1**	**0.075**	**0.785**
MS × TNBS	**1**	**0.072**	**0.789**
Sex × MS × TNBS	**1**	**0.631**	**0.429**
Residuals	**102**		
(E) Total travel distance			
Sex	**1**	**8.243**	0.005*
MS	**1**	**4.379**	0.038*
TNBS	**1**	**1.089**	**0.298**
Sex × MS	**1**	**0.677**	**0.412**
Sex × TNBS	**1**	**3.180**	**0.077**
MS × TNBS	**1**	**0.901**	**0.344**
Sex × MS × TNBS	**1**	**2.867**	**0.092**
Residuals	**102**		

Results of 3-way ANOVA of time spent in open arm (A), % time spent in open arm (B), % open arm entries (C), and time spent in closed arm (D), total travel distance (E) with sex, maternal separation (MS), and previous inflammation (TNBS) as 3 factors were shown. ^*^P < 0.05, ^**^P = 0.001, and ^***^P = 0.0001

### Neuroendocrine function

#### Plasma ACTH

The results of the plasma ACTH concentration are presented in Table 2. Table 2 shows the results of a three-way ANOVA of plasma ACTH, indicating a significant sex difference in plasma ACTH. In the basal condition, plasma ACTH was shown to have a significant sex effect (F_1.51_ = 14.488, *p* < 0.0001), sex × MS interaction (F_1.51_ = 4.721, *p* = 0.034), and MS × TNBS interaction (F_1.51_ = 8.541, *p* = 0.005) with a trend interaction of sex × MS × TNBS (F_1.51_ = 3.333, *p* = 0.074). After the CRD, plasma ACTH showed a significant sex × MS × TNBS interaction (F_1.51_ = 8.418, *p* = 0.006).

**Table 2:** Three-way ANOVA results of plasma ACTH and serum corticosterone levels

Effects	df	F	*P*-level
no-CRD ACTH			
Sex	**1**	**14.488**	0.0001***
MS	**1**	**1.462**	**0.232**
TNBS	**1**	**3.422**	**0.07**
Sex × MS	**1**	**4.721**	0.034*
Sex × TNBS	**1**	**2.611**	**0.112**
MS × TNBS	**1**	**8.541**	0.005*
Sex × MS × TNBS	**1**	**3.333**	**0.074**
Residuals	**51**		
no-CRD corticosteron			
Sex	**1**	**130.7**	0.0001***
MS	**1**	**0.112**	**0.739**
TNBS	**1**	**2.366**	**0.13**
Sex × MS	**1**	**1.501**	**0.226**
Sex × TNBS	**1**	**0.322**	**0.573**
MS × TNBS	**1**	**6.843**	0.012*
Sex × MS × TNBS	**1**	**1.708**	**0.197**
Residuals	**52**		
CRD ACTH			
Sex	**1**	**1.672**	**0.201**
MS	**1**	**0.194**	**0.661**
TNBS	**1**	**0.198**	**0.659**
Sex × MS	**1**	**1.833**	**0.182**
Sex × TNBS	**1**	**0.214**	**0.646**
MS × TNBS	**1**	**0.672**	**0.416**
Sex × MS × TNBS	**1**	**8.418**	0.006*
Residuals	**47**		
CRD corticosterone			
Sex	**1**	**35.862**	0.0001***
MS	**1**	**0.329**	**0.569**
TNBS	**1**	**1.253**	**0.269**
Sex × MS	**1**	**0.069**	**0.794**
Sex × TNBS	**1**	**0.043**	**0.836**
MS × TNBS	**1**	**0.43**	**0.515**
Sex × MS × TNBS	**1**	**0.329**	**0.569**
Residuals	**48**		

Results of 3-way ANOVA of neuroendocrine data with or without colorectal distention (CRD) with sex, maternal separation (MS), and previous inflammation (TNBS) as 3 factors were shown. ^*^P < 0.05, ^**^P = 0.001, and ^***^P = 0.0001

**Fig. 7:**
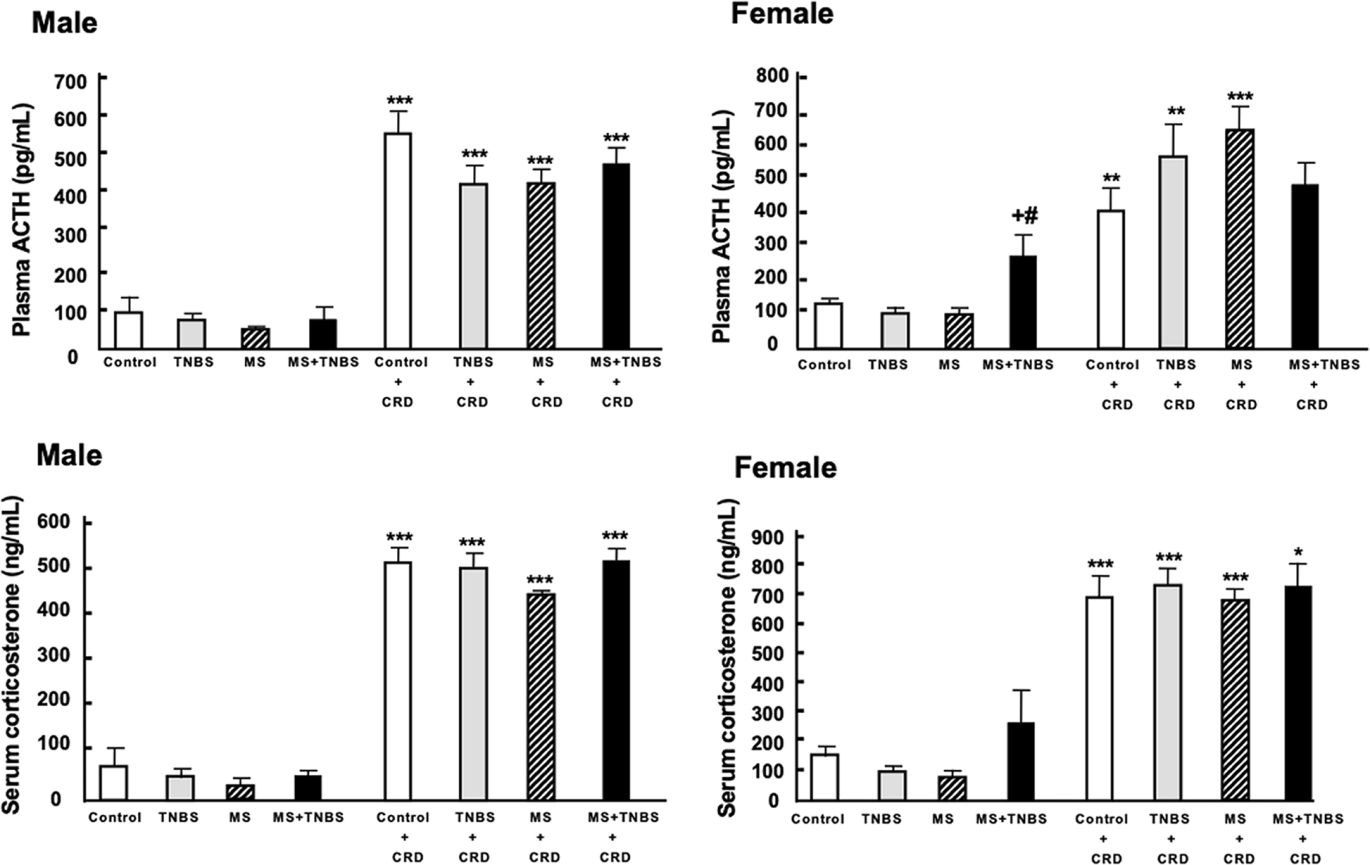
Plasma ACTH and serum corticosterone levels. All data are expressed as the mean ± SE (*n* = 6–10). Baseline (without colorectal distention [CRD]) and post-CRD values are shown. ^*^P < 0.01, ^**^P < 0.007, ^***^P < 0.0001 vs. same treatment (post hoc test following ANOVA). ^#^*P* < 0.001 MS + TNBS vs. TNBS, ^+^*P* < 0.05 MS + TNBS vs. MS (female rats)

In male rats, there was no significant difference in plasma ACTH among controls (*n* = 6), TNBS (*n* = 6), MS (*n* = 9), and MS + TNBS (*n* = 7) at baseline. There was no significant difference in plasma ACTH in rats after the CRD (control + CRD, *n* = 6; TNBS + CRD, *n* = 6; MS + CRD, *n* = 10; and MS + TNBS + CRD, *n* = 8). ANOVA revealed a significant main effect of CRD (F_1.49_ = 258.421, *p* < 0.0001) and a MS × TNBS interaction (F_1.49_ = 5.277, *p* < 0.026) (Fig. 7). In male rats, CRD induced a significantly different increase in plasma ACTH in controls (94.3 ± 30.7 pg/mL to 559.7 ± 53.2 pg/mL, *p* < 0.0001), TNBS (76.1 ± 13.4 pg/mL to 428.8 ± 47.9 pg/mL, *p* < 0.0001), MS (50.7 ± 6.3 pg/mL to 429.1 ± 32.9 pg/mL, *p* < 0.0001), and MS + TNBS (78.9 ± 24.1 pg/mL to 480.1 ± 49.8 pg/mL, *p* < 0.0001, post hoc test) (Fig. 7).

On the other hand, female rats had a different plasma ACTH pattern among the groups (*n* = 6–10; Fig. 7). ANOVA revealed significant main effects of CRD (F_1.49_ = 86.295, *p* < 0.0001) and a MS × TNBS interaction (F_1.49_ = 10.590, *p* < 0.002). In female rats, CRD induced significant increases in plasma ACTH in controls (133.3 ± 13.7 pg/mL to 410.2 ± 65.2 pg/mL, *p* < 0.007), TNBS (106.2 ± 12.1 pg/mL to 573.5 ± 97.7 pg/mL, *p* < 0.005), and MS (104.4 ± 12.4 pg/mL to 651.1 ± 72.3 pg/mL, *p* < 0.0001). ANOVA of the plasma ACTH of female rats at baseline showed a significant difference among the groups (F_3,27_ = 5.186, *p* < 0.006). Female rats with MS + TNBS at baseline had a significantly higher plasma ACTH (278.3 ± 75.2 pg/mL) than those with TNBS (106.2 ± 12.1 pg/mL, *p* = 0.008) or MS (104.4 ± 12.4, *p* < 0.011, post hoc test). Therefore, CRD did not cause a significant increase in plasma ACTH in female rats with MS + TNBS.

#### Serum corticosterone

The results of the serum corticosterone concentration are presented in Fig. 7. Table 2 shows the results of three-way ANOVA of serum corticosterone, indicating a significant sex difference in serum corticosterone levels. In the basal condition, serum corticosterone was revealed to have a significant sex effect (F_1.52_ = 130.7, *p* < 0.0001) and MS × TNBS interaction (F_1.52_ = 6.843, *p* = 0.012). After the CRD, serum corticosterone showed a significant sex effect (F_1.48_ = 35.862, *p* < 0.0001).

The changes in the serum corticosterone of male rats were essentially similar to those seen for plasma ACTH (*n* = 6–10; Fig. 7). ANOVA revealed a significant main effect of CRD (F_1.49_ = 777.245, *p *< 0.0001). There was a trend interaction between MS × TNBS in serum corticosterone (F_1.49_ = 3.715, *p* < 0.060). CRD induced a significant increase in serum corticosterone in controls (75.2 ± 40.4 ng/mL to 531.9 ± 32.8 ng/mL, *p* < 0.0001), TNBS rats (55.1 ± 19.4 ng/mL to 521.3 ± 31.5 ng/mL, *p *< 0.0001), MS rats (30.4 ± 2.6 ng/mL to 462.5 ± 14.6 ng/mL, *p* < 0.0001), and MS + TNBS rats (54.5 ± 15.3 ng/mL to 534.5 ± 27 ng/mL, *p* < 0.0001, post hoc test).

The changes in the serum corticosterone of female rats were also similar to those in plasma ACTH (*n* = 6–10; Fig. 7). ANOVA revealed significant main effects of CRD on serum corticosterone (F_1.50_ = 176.051, *p* < 0.0001). In female rats, CRD induced a significant increase in serum corticosterone in controls (155.9 ± 37.1 ng/mL to 698.0 ± 75.9 ng/mL, *p* < 0.0001), TNBS (98.6 ± 17.7 ng/mL to 739.8 ± 59.4 ng/mL, *p* < 0.0001), MS (81.4 ± 24.2 ng/mL to 684.8 ± 34.6 ng/mL, *p* < 0.0001), and MS + TNBS (271.6 ± 112.5 ng/mL to 732.1 ± 94.7 ng/mL, *p* < 0.01).

## Discussion

### Hyperactive behaviors dominantly in male animals after MS + TNBS

This is the first study to show that a combination of early-life stress and gut inflammation may specifically lead to hyperactive behaviors in male animals. The EPM is believed to be a task that evaluates animals exposed to unconditioned fear or the anxiolytic properties of drugs [44, 63]. However, the idea that an increased presence of animals in the open arms of the EPM can be used as an index of impulsivity has been proposed by Ueno et al. [64]. There are several distinct animal models of ADHD, including spontaneously hypertensive rats (SHRs) [65] and stroke-prone spontaneously hypertensive (SHRSP) rats [66]. SHR and Wistar Kyoto rats display less anxiety-related behavior in the EPM than Sprague–Dawley rats [67]. SHRSP rats display higher motor activity, impulsivity, inattention, and dopaminergic hypofunction and decreased 5-hydroxytryptamine in the brain than SHRs [64]. Entries into open arms in the EPM task are also remarkably increased in SHRSP rats [64]. The percentage of time spent in open arms by male rats with MS + TNBS in this study (37.4 ± 3.4%) is higher than that of SHRSP rats in the report of Ueno et al. [64]. Therefore, it is natural to assume that a combination of early-life stress and gut inflammation may be one of the risk factors for comorbid ADHD and IBS in males.

### Relevance of hyperactive behaviors and combination of recovered gut inflammation and early-life stress in animals and humans

The male:female sex ratio in ADHD children is approximately 9:1 [68, 69]. Inherent or genetic components related to the Y chromosome probably play important roles in the pathogenesis of ADHD [70]. It is of great interest to note distinct abnormal behavior predominantly in males after combined early trauma and gut inflammation. In even SHRSP rats, the most widely used model of ADHD, female rats also spend more time in open arms [64]. Therefore, at least for time spent in open arms, early trauma combined with gut inflammation seems to better reflect the features of hyperactive behavior. We originally expected that the combination of early trauma and gut inflammation in female rats would induce anxiety-like behavior, which is well known to be comorbid with IBS [34]. However, ADHD is also comorbid with IBS [35]. We previously reported that IBS patients show hypoactivity of the right dorsolateral prefrontal cortex during tasks needed cognitive flexibility [71]. There is a report that children with right hemisphere damage or dysfunction show symptoms of ADHD [72]. Therefore, the combination of early trauma and previous colonic inflammation may synergistically impair inhibitory function of the forebrain against the limbic brain in male animals, resulting in hyperactive behavior.

### Dysregulation of homeostasis in rats after MS + TNBS

Hyperactive behavior and anxiety/depression are not mutually exclusive phenomena. The rate of comorbid psychiatric and learning problems, including anxiety and depression, ranges from 12 to 60% in patients with ADHD [52]. Both increased (hyperactive) and decreased (anxiety) time spent in the open arms of the EPM imply externally oriented and dysregulated non-homeostatic behaviors. Thus, an elevated basal plasma ACTH in female rats exposed to MS + TNBS may reflect interoception-oriented dysregulation of homeostasis. This may in part explain the female predominance of IBS in western countries with high levels of stress.

### HPA axis in female rats after MS + TNBS

Early trauma [25] or previous gut inflammation [37] increases IBS risk and IBS patients show elevated ACTH [41]. In addition, elevated plasma ACTH correlates well with plasma interleukin-6 in IBS patients [73]. Neonatal rats exposed to MS also demonstrate exaggerated activation of the HPA axis [47, 48]. Reduced expression of hippocampal glucocorticoid receptors caused by negative feedback to the paraventricular nucleus of the hypothalamus [57] due to DNA methylation [58, 74] has been shown to be one of the causes of elevated plasma ACTH. Shanks et al. [75] reported that exposure of neonatal rats to a low dose of endotoxin causes long-lasting changes in the activity of the HPA axis and an elevated mean serum corticosterone concentration due to increased corticosterone pulse frequency and amplitude. Moreover, estrogen and progesterone likely play some sex role in the mechanism of IBS [49]. Because elevated plasma ACTH was selectively seen in the female rats of our study, the same mechanism is suggested to alter the HPA axis.

### HPA axis in male rats after MS + TNBS

In the present study, the same levels of plasma ACTH and corticosterone were detected in male rats exposed to early trauma, previous inflammation, or their combination at baseline or at visceral stimulation. In contrast, an earlier study [74] showed an increased ACTH and corticosterone response to restraint stress in male rats treated by a low-licking mother. We cannot simply compare our data with those of earlier studies because early experience and gut manipulation are different each other. CRH and HPA axis function are not always parallel to exploratory behavior [44]. Therefore, our present data are not surprising and we cannot rule out the potential role of the CRH system in stress response and behavior after MS + TNBS in male rats.

### Strength

There are some strengths in this study. The first strength is that both male and female rats were examined. Most previous studies were limited to male rats. However, female rats seem to be apparent hyperactive more frequently than male rats. We investigate hyperactive behaviors through a method directly compared with behaviors in male control rats and female control rats. These phenomena may reflect alternate anxiety symptoms in female rats, as discussed above, but the precise mechanism needs to be addressed. Second, the menstrual cycles of the female rats were precisely determined. Because visceral sensation and emotion depend on the menstrual cycle [49, 50, 60], the influence of this cycle on the results was excluded.

### Limitation

There are some limitations to this study. The first limitation is that neither anxiety-like behaviors in female rats nor deactivation of the HPA axis in male rats after CRD was observed after MS + TNBS. Rather than that, plasma ACTH response to CRD was blunted in female rats after MS + TNBS. This is partially different from original hypothesis. However, it is concordant with basal increase in plasma ACTH in female rats after MS + TNBS and an earlier study with blunted responses of the HPA axis after chronic stress [76]. The second limitation is that molecular events inside the rats’ brains could not be examined. The detection of dopaminergic function or the 5-hydroxytryptaminergic molecule as well as modification of ADHD-like behavior using methylphenidate [64] is the future direction of this model. Third, the immune function of the gut has not yet been determined. In addition, whether these phenomena are specific to gut inflammation or found in other organs is unknown. A recent study indicated that immature rats with early-life stress have impaired intestinal barrier function that permits increased penetration of antigens and/or microbeads into the body [32]. They also reported that CRH antagonists may play a role in the colonic mucosal changes induced by MS. Visceral afferent nerve terminals have been shown to be sensitized by degranulated mast cells after acute stress [36]. Additionally, activation of enterochromaffin cells has also been suggested to play a role in this sensitization [31]. Exposure of neonatal rats to endotoxin has long-lasting effects on immune system regulation, including increased sensitivity of lymphocytes to stress-induced suppression of proliferation and remarkable protection against adjuvant-induced arthritis. Collins et al. [39] reported that stress caused a greater increase in myeloperoxidase activity in rats administered TNBS for 6 weeks than in controls, even though the plasma corticosterone levels were similar between the two groups. Moreover, previous colitis renders the colon more susceptible to the effects of stress on enteric nerve function and increases inflammation parameters in response to stress [39]. Therefore, molecular and immunological changes in the gut as well as the specificity of previous gut inflammation on behavioral or neuroendocrine changes should be addressed in a future study.

## Conclusions

Our hypothesis that MS and gut inflammation synergistically induce hyperactivity in male rats but not in female rats was supported by our findings. In contrast, MS and gut inflammation synergistically induce exaggerated secretion of plasma ACTH after CRD in female rats. This study supports the hypothesis that there is a potent and long-lasting effect of neonatal exposure that can program major changes in the development of neuroendocrine, immunological regulatory, and behavioral characteristics.

## Data Availability

Datasets are available upon reasonable request.

## Abbreviations

ACTH: Adrenocorticotropic hormone
ADHD: Attention-deficit/hyperactivity disorder
CRD: Colorectal distention
EPM: Elevated plus-maze
IBS: Irritable bowel syndrome
MS: Maternal separation
PND: Post-natal day
TNBS: Trinitrobenzene-sulfonic acid
